# Hypertrophy of the Prostate Gland

**Published:** 1891-01-10

**Authors:** 


### SURGERY.

### HYPERTROPHY OF THE PROSTATE GLAND.

A recent surgical author states that hypertrophy of the prostate gland never occurs before the age of fifty-four, and is most common from fifty-seven to sixty. Sir Henry Thompson has never met with a case before the age of fifty-four. It is also stated that if it does not occur before sixty-five, it very rarely happens. The causes of the change are unknown. It consists in an excess of development in the unstriated muscular fibres, connective tissue, and granular tubes and crypts which compose the organ. Most commonly the whole gland is involved, sometimes only a portion. Mr. Harrison some time back made the suggestion that the prostate is a muscular organ supporting and aiding the natural retention of the contents of the bladder. Hence it is called into requisition more in advancing years as muscular activity diminishes, taking on a condition of hypertrophy. Mr. Harrison also remarked in the *British Medical Journal* of last year that the term fibromatous prostate seems to represent better than any other that condition of the part most frequently coming under the attention of the surgeon. The muscular prostate, he thinks, is capable of adapting itself to circumstances when a fibrous one is not. A large prostate, he thinks, may functionally mean very little. When, however, the enlargement is chiefly fibromatous, the liability to the accession of urine symptoms is greatly increased. Some exception, however, has been taken to Mr. Harrison's views by Dr. Joseph Griffiths, of the Surgical Department, Cambridge. The latter does not think that the prostate acts as a support to the bladder, for the gland at the time of birth is merely a small mass behind the urethra, and urine is retained long before it is possible for the prostate to act as a sphincter. Moreover, there are instances when the gland remains in a rudimentary state throughout life. In the same journal of last year, Mr. Harrison related some cases in practice bearing on the functions of the prostate, by which he endeavours to support the views previously brought forward.

What, however, the generality of practitioners desire to know is the best treatment for an enlarged prostate. The first and most important effect of a large prostate is obstruction to the flow of urine from the secondary resulting changes in the course and length of the urethra. This causes diminution in the force of the stream, increased frequency of micturition, fullness and uneasiness about the perinæum, and ultimately chronic cystitis. The difference between the symptoms of enlarged prostate and stricture of the urethra are principally that in the first case the stream of urine is little diminished in size, whereas in stricture it is small. In the first case straining does not increase the flow, while in stricture it does. Unfortunately, the treatment of enlarged prostate is palliative only. The patient must avoid irregular habits, exposure to cold, and fatigue. But the chief means are instrumental, and they cannot be commenced too soon. As a general rule, the regular use of the catheter twice a-day is required. Many think the best instrument for use is a French coudèe, No. 8 or 9. If this does not answer a *catheter à boule* may be tried. Occasionally a silver prostatic catheter is necessary, four inches longer than an ordinary catheter, with its curved portion a third of a circle five and a-half inches in diameter, the handle to be well depressed. The above are the ordinary recommendations given in surgical text-books. But in the *British Medical Journal* of May 17th last, Mr. Harrison publishes an article headed "On the Selection and Use of Catheters and other Instruments for Enlarged Prostate." Mr. Harrison observes that when he first entered the profession, the instrument most commonly employed was the long curved silver catheter. But this has now been largely discarded, and has given way to less dangerous implements, in the use of which skill and gentleness take the place of the force which even the sight of this weapon seems to imply. Mr. Harrison advises the use of flexible catheters, which are readily introduced and rarely cause hæmorrhage. Those made of silk web have some advantage over the rubber catheters, as they can be used with a little more firmness. But in one case a coudèe slips in easiest, in another an olive-pointed instrument, and in a third an ordinary round-headed catheter. "It is important that the delivery power of the instrument should be relative to the normal size of the urethra, and not greatly in excess." For it is deleterious, if the urine is drawn off *pleno rivo*, when a person has been accustomed to ooze or dribble his urine. Too rapid emptying of the bladder in the regular employment of catheterism is an important factor in producing entire loss of natural expulsive power, for "nature will not provide muscle when it is not wanted." Moreover, when a bladder has been accustomed to contain some few ounces of urine, the sudden withdrawal establishes a condition of flaccidity of the vesical walls which is favourable to the admission of air either by the instrument or by the urethra. There is also an excessive transudation of mucus; and a sanguineous discharge may take place from the vesical walls suddenly deprived of their support. In this way material for decomposition is provided, and so-called catheter fever is set up. Where the urine is acid it should be removed by instalments, and where alkaline or unhealthy it should be replaced by some antiseptic fluid. Morris also has given very good advice with regard to the use of the catheter. He says: To prevent the occurrence of hæmorrhage great care should be taken before the first catheterism. The catheter should never be used on a first occasion upon a person while standing. Neither coughing nor straining on the part of the patient, nor hypogastric pressure by the surgeon's hand should be permitted. To this it may be added that a small dose of opium before a first catheterism is often beneficial. Most frequently habitual catheterisation by the patient himself becomes a necessity. This should not, however, be allowed until he has become well used to the presence of instruments. The danger of urinary fever should also be explained to him, also of hæmorrhage, which may be followed by cystitis and pyelitis.

One of the complications of enlarged prostate is an attack of urethral spasm. For example, a person suffers from enlarged prostate, and suddenly spasms occur, the pain extending from the prostate to the glans, and causing complete inability to micturate. When such a case occurs Mr. Fraser Stokes, of Highbury, suggests it would be advisable to place the patient under an anæsthetic, and carefully sound, when probably a calculus would be found lying behind the prostate. But H. M. M., a medical man, writes as himself as a sufferer (*British Medical Journal*, March, 1889), when suffering from spasms from prostate to glans, as if a red-hot wire were drawn throughout, he has found the greatest relief by drawing off the urine and washing out the bladder with soft warm water. Mr. W. S. Elliott advises morphine injections hypodermically in such cases, and details a case when this practice was most successful. Other means used are henbane, alkalies, senega, and morphine suppositories.

The treatment of retention from prostatic enlargement was discussed at the last meeting of the British Medical Association at Leeds, Mr. McGill, Professor of Surgery in the Yorkshire College, opening the discussion. His propositions were, that prostatic enlargements which give rise to urinary symptoms are intra-vesical and not rectal. That retention is caused by a valve-like action of the intra-vesical prostate. That in many cases self catheterism is the only treatment required.

But in advanced cases of prostatic disease, the patient's life becomes a burden to him; the bladder is utterly intolerant of urine, and the catheter must be passed every hour, or oftener. Various suggestions have been made for the relief of these miserable sufferers. Sir Henry Thompson's operation consists in making an incision above the *symphysis pubis* on the end of a large hollow sound, previously passed into the bladder. An elastic catheter is then introduced, through which the urine passes. Another method is making a perinæal incision on a grooved staff, and inserting an india-rubber tube. A third is tunnelling the large prostate, as introduced by Mr. Harrison. The operation consists in puncturing the bladder with a trocar about one inch in front of the anus. A fourth method is to follow the steps in lithotomy, and incise the prostate on both sides (prostatotomy). Lastly, Mr. McGill has removed portions of a large prostate with scissors through a supra-pubic opening. At the discussion at Leeds referred to above, a table was given of supra-pubic prostatectomies, performed in the Leeds General Infirmary up to July '89. In seven cases out of twenty-four, lithotomy was also necessitated. This is less than 30 per cent., and scarcely bears out the view referred to as entertained by Mr. Stokes, that spasmodic attacks are usually due to the complication of a calculus in the bladder.

Mr. McGill states that when the catheter treatment fails, radical treatment is necessary; that radical treatment should for a time thoroughly drain the bladder, and permanently remove the cause of the obstruction; that these two last indications are best fulfilled by a supra-pubic operation.

In conclusion, it may be remarked that Mr. Hugh Woods, of Highgate, has recently written that in many cases medicinal treatment is sufficient, and he recommends liquid extract of ergot. The mixture which he uses contains in each drachm, liquid extract of ergot, m. 7½; tinct. of belladonnæ and liq. morphiæ, of each m. 3¾; with sulphate of magnesia. He has repeatedly found this mixture efficacious in relieving retention of urine in cases of enlarged prostate, obviating the use of the catheter with its attendant risks, and removing all urgent symptoms for a considerable period. It should be given in ʒi doses in water. The formula is prepared by Messrs. Willows and Francis, 101, High Holborn. Dr. Woods thinks there are a large class of cases of enlarged prostate, in which medicinal treatment is decidedly preferable to the use of the catheter, or other surgical interference. Nevertheless, the time often arrives when surgical treatment becomes a necessity.

